# Betacoronavirus Infection Outbreak, São Paulo, Brazil, Fall 2023

**DOI:** 10.3201/eid3003.230990

**Published:** 2024-03

**Authors:** Tânia do Socorro Souza Chaves, Ana H. Perosa, Gabriela Barbosa, Diogo B. Ferreira, Nancy Bellei

**Affiliations:** Universidade Federal do Pará, Belém, Brazil (T.D.S.S. Chaves);; Universidade Federal de São Paulo, São Paulo, Brazil (T.D.S.S. Chaves, A.H. Perosa, G. Barbosa, D.B. Ferreira, N. Bellei)

**Keywords:** betacoronavirus, coronavirus, human coronavirus, viruses, COVID-19, outbreak, respiratory infections, severe acute respiratory syndrome coronavirus 2, SARS-CoV-2, SARS, coronavirus disease, zoonoses, viruses, São Paulo, Brazil

## Abstract

We report a human coronavirus OC43 infection outbreak in hospitalized patients and healthcare workers in São Paulo, Brazil, occurring after SARS-CoV-2 cases disappeared. Infection was associated with healthcare workers in 5 (29.4%) patients. Routine surveillance including a respiratory virus panel can improve coronavirus detection in both healthcare professionals and patients.

The COVID-19 pandemic has caused major human and social behavior changes. Human coronavirus (HCoV) OC43, a common human coronavirus, remains a major cause of respiratory infections. HCoV-OC43 can infect humans at any age, causing lower respiratory tract infections that can be severe in patients who have concurrent conditions (*1*,*2*). Until May 2023, a total of 2,533 patients were hospitalized with COVID-19 at Hospital São Paulo (São Paulo, Brazil). We report an unexpected HCoV-OC43 infection outbreak among patients and healthcare workers at Hospital São Paulo. We conducted this observational study in compliance with institutional guidelines and approval by the Ethics Committee of Universidade Federal de São Paulo (CEP/UNIFESP no. 29407720.4.00 00.5505). 

During March–June 2023 (fall season), we collected swab specimens from patients and screened those specimens for influenza A/B virus, respiratory syncytial virus, SARS-CoV-2, and HCoV in the laboratory at our hospital as a routine surveillance method used since 2020. We evaluated samples from 927 persons who had acute respiratory infections: 446 hospitalized patients and 481 healthcare workers. We detected HCoV by using multiplex real-time PCR with specific primers and probes for HCoV-OC43, HCoV-229E, HCoV-40 HKU-1, and HCoV-NL63 (*3*,*4*). Among tested samples, 7.7% (71/927) were positive for HCoV: 10.6% (51/481) for healthcare workers and 4.5% (20/446) for hospitalized patients (Table).

**Table:** HCoV-positive case-patients by month, age, and participant groups during the betacoronavirus infection outbreak in Hospital São Paulo, São Paulo, Brazil, March–June 2023*

Characteristic	Total	Hospitalized patients	Healthcare workers
Total	71/927 (7.7)	20/446 (4.5)	51/481 (10.6)
March	4/295 (1.3)	2/128 (1.6)	2/167 (1.2)
April	5/195 (2.6)	2/104 (1.9)	3/91 (3.3)
May	28/218 (12.8)	4/102 (3.9)	24/116 (20.7)
June	34/219 (15.5)	12/112 (10.7)	22/107 (20.6)
Adults (>12 y old)	63/813 (7.7)	12/332 (3.6)	51/481 (10.6)
Children	8/114 (7.0)	8/114 (7.0)	NA

*Values are no. positive/no. tested (%). HCoV, human coronavirus; NA, not applicable.

Of the 71 HCoV-positive samples, 28.2% (20/71) were obtained from hospitalized patients (mean age 34.5 years; interquartile range 6–64 years) and 71.8% (51/71) from healthcare workers (mean age 41.9 years; interquartile range 32–52 years). Among healthcare workers, 46 (90.2%) samples were positive for HCoV-OC43, 4 (7.8%) for HCoV-NL63, and 1 (2%) for HCoV-229E. Among hospitalized patients, 16 (80%) patients were positive for OC43, 3 (15%) for NL63, and 1 (5%) for HKU-1. Co-infections were identified in only 4 (5.6%) case-patients: 1 patient had both HCoV-NL63 and SARS-CoV2, 1 patient had both HCoV-OC43 and respiratory syncytial virus, and 2 patients each had both HCoV-OC43 and influenza A(H1N1)pdm09 virus.

All 16 inpatients who had HCoV-OC43 had risk factors for more severe illness, such as immunosuppression (3 patients) and underlying conditions (8 patients); 5 (31.2%) patients had both. Two (2/16; 12.5%) immunosuppressed patients required admission to an intensive care unit and died (1 child, 1 adult).

Radiologic images were obtained for 14 of 16 inpatients who had HCoV-OC43, and 62.5% (10/16) had an alteration detected by chest computed tomography. Radiologic findings included lung opacities, bilateral interstitial infiltrate, consolidations, and centrilobular micronodules with a unifocal or multifocal ground glass pattern, all of which were predominantly distributed within the lower lobes.

A probable nosocomial acquisition might have occurred because the infection rate among healthcare workers peaked earlier (May) than the observed inpatient peak rate (June) (Table). Five (31.2%) inpatients who had HCoV-OC43–positive samples were housed within different wards several days after admission; thus, it was possible to confirm nosocomial acquisition. In those cases, HCoV-OC43 transmission took place within inpatient wards, specifically during activities involving direct contact with a healthcare worker. Contact tracing connected patient cases to interactions with healthcare workers.

The SARS-CoV-2 positivity rate during the outbreak period (March–June) varied from 7.3% to 27.9% (Figure); no cases were reported in June. To provide background for the outbreak, we conducted surveillance testing for respiratory viruses collected 2 months before (January–February) and after (July–August) the outbreak; no HCoV was detected during those periods. In previous studies conducted at our hospital before the COVID-19 pandemic, we did not observe >5% monthly circulation of HCoVs during the study years under investigation (*5*). The HCoV-OC43 outbreak peak occurred after the disappearance of SARS-CoV-2 cases (Figure).

**Figure:**
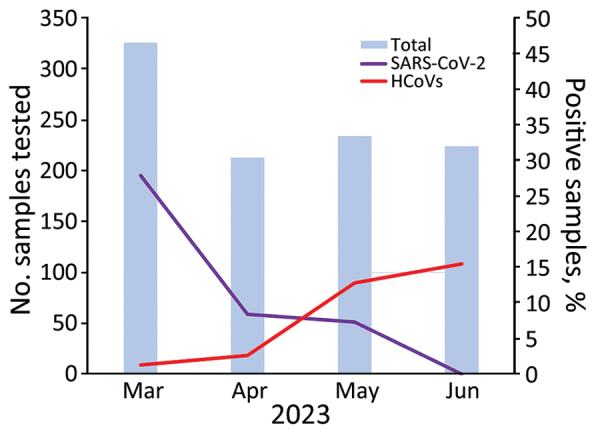
Positivity rates for SARS-CoV2 and HCoV in Hospital São Paulo during betacoronavirus infection outbreak, São Paulo, Brazil, March–June 2023. Blue bars indicate the number of samples tested each month. Purple and red lines indicate the percentage of samples that were positive for each virus. HCoV, human coronavirus.

In this outbreak, hospitalized patients showed evolution of a severe form of infection caused by HCoV-OC43. Nirmatrelvir/ritonavir is a promising antiviral drug combination in preclinical studies that inhibits the proteolytic activity of SARS-CoV-2 M^pro^, a cysteine protease found in the family Coronaviridae (*6*), and might be useful for treating HCoV-OC43 infections.

At the end of March 2023 in Brazil, the National Health Surveillance Agency (Agência Nacional de Vigilância Sanitária) updated guidelines for mask use in healthcare settings. Since April 2023, the hospital committee has relaxed the requirement for universal mask use, making them obligatory only in areas designated for patient care (*7*). Relaxing mask use by healthcare workers who provide care to high-risk patients likely contributed to nosocomial acquisition of HCoV-OC43. Furthermore, healthcare workers who had respiratory infections other than SARS-CoV-2 infections were likely less vigilant in using personal protective equipment during patient care. In addition, an unconscious relaxation in maintaining precautions might have occurred, possibly because persons did not perceive themselves as potential transmitters.

In conclusion, we report the occurrence of HCoV-OC43 causing severe acute respiratory infection that might be underestimated because of a lack of better diagnostic approaches for viral respiratory infections, particularly in high-risk patients. Routine surveillance using a diagnostic panel of respiratory viruses can improve detection in both healthcare workers and patients and can help determine prevalence and prevent transmission of different viruses.

## References

[1] Chow EJ, Uyeki TM, Chu HY. The effects of the COVID-19 pandemic on community respiratory virus activity. Nat Rev Microbiol. 2023;21:195–210. 36253478 10.1038/s41579-022-00807-9 PMC9574826

[2] Vabret A, Mourez T, Gouarin S, Petitjean J, Freymuth F. An outbreak of coronavirus OC43 respiratory infection in Normandy, France. Clin Infect Dis. 2003;36:985–9. 10.1086/374222 12684910 PMC7109673

[3] Kesheh MM, Hosseini P, Soltani S, Zandi M. An overview on the seven pathogenic human coronaviruses. Rev Med Virol. 2022;32:e2282. 10.1002/rmv.2282 34339073

[4] Dare RK, Fry AM, Chittaganpitch M, Sawanpanyalert P, Olsen SJ, Erdman DD. Human coronavirus infections in rural Thailand: a comprehensive study using real-time reverse-transcription polymerase chain reaction assays. J Infect Dis. 2007;196:1321–8. 10.1086/521308 17922396 PMC7109921

[5] Cabeça TK, Granato C, Bellei N. Epidemiological and clinical features of human coronavirus infections among different subsets of patients. Influenza Other Respir Viruses. 2013;7:1040–7. 10.1111/irv.12101 23462106 PMC4634278

[6] Owen DR, Allerton CMN, Anderson AS, Aschenbrenner L, Avery M, Berritt S, et al. An oral SARS-CoV-2 M^pro^ inhibitor clinical candidate for the treatment of COVID-19. Science. 2021;374:1586–93. 10.1126/science.abl4784 34726479

[7] ANVISA. Technical Note GVIMS/GGTES/ANVISA No.04/2020. Guidelines for health services: prevention and control measures that must be adopted when providing care to suspected or confirmed cases of COVID-9 infection [in Portuguese] [cited 2024 Jan 3]. https://www.gov.br/anvisa/pt-br/centraisdeconteudo/publicacoes/servicosdesaude/notas-tecnicas/2020/nota-tecnica-gvims_ggtes_anvisa-04_2020-25-02-para-o-site.pdf

